# Extracts

**Published:** 1883-03

**Authors:** 


### Extracts.

---

INFANTILE PARALYSIS.—It is almost unnecessary to observe that the subject of infantile paralysis is one of those of which our clinical and pathological knowledge has recently been greatly improved, and certainly the treatment of which is more scientific than it was only a few years ago.

I am inclined to think, however, that we have it in our power to benefit cases of this affection to a greater extent than is generally supposed, and that we may save parents much trouble and anxiety by instructing them in the observance of certain simple details, the neglect of which may produce serious consequences to their children.

It is doubtless within the experience of most of us, both in hospital and in private practice, to find that parents are extremely solicitous about a child that is affected with this form of paralysis; and we can understand how natural must be the desire to avert, as far as possible, the disadvantages which, in future life, are imposed upon those who are afflicted with any serious bodily defect.

In looking back over ten years' experience at a children's hospital, it appeared to me that, in regard to the treatment of infantile paralysis, I had neglected to give the subject all the attention it deserved, until a few months since, when a case of this kind (a girl twelve years of age) came to the hospital, eight years having elapsed since she was first brought under my care.

It is a matter of regret to me that, in a total of rather more than one hundred cases of this malady, entered in my note-book, there is little or no attention paid to the points which, I now perceive, are of most importance. The respective ages, the limb or limbs affected, the difference of temperature, degree of wasting, and muscular irritability or reaction to electrical stimuli—these are noted; but, after a period of a few weeks, the histories of the cases come to an end, and the patients are lost sight of. Generally, and at one time invariably, I followed the common plan of transferring the cases to the special department in which electro-galvanism, or some other form of electro-therapy, was administered.

In private practice, we have this advantage, that we are able to watch the patients for long periods—difficult in the case of hospital patients, unless some particular plan is adopted to keep them under notice.

The mother of the girl referred to reminded me that she had promised to bring her daughter when she was twelve years of age; and the intelligent account she was able to give led me to reflect on what might be best done for similar cases. When first seen, this was a typical example of paralysis of the left leg, of such a date, with such a wasting, as to justify the belief that there would be serious permanent difference between the limbs. The chief means by which this had been prevented was the great attention given by the mother to keeping up the temperature of the affected limb. Daily, for eight years, night and morning, the leg had been bathed in hot water for an hour at a time. During the day it was carefully covered with wool; and, during the night, heat was obtained by means of hot water in bottles. The girl walked with a limp, but there was no difference in size between the healthy and affected limb. In length and circumference they were equal. The leg was paralyzed below the knee; the foot was everted; and the lower part of the limb was moved with a slight swing, as is commonly observed in such cases. The state of nutrition of the tissues of the leg was such as to suggest the question whether there might not be more advantage in the method of treatment, simple as it had been, which was followed in his case, than when electro-therapy is employed.

I am under the impression that the results obtained, in the special department of our hospital, where cases were treated by electro-therapy, were not so satisfactory as to make us attach great importance to it; and I am inclined to think, when the excitement and terror which arose in some cases were considered, positive harm was done.

I am anxious to press this point on your attention, because there is a general belief that little or no good can be done to these cases without electro-therapy; and poor children who cannot obtain it are left to suffer many troubles, which could be prevented by proper directions given to parents in regard to details simple in themselves, but, when continued for several years, of great permanent effect and benefit.

It is true, apparently, that in cases where no attention has been given to the child for several months after the primary attack, the electric stimulation of wasted muscles, as is well known, on two or three occasions, may be beneficial; but this is different in its object from the frequent and repeated use of galvanism. I mean to say that if we intend in using the battery simply to prevent the wasting which marks the disease, assumedly due to defective nervous action or stimulus, shall we not obtain the same physiological effects by following the treatment which had such good results in the case of the girl I have mentioned?

The question which is suggested is this: Have we not in artificial heat distinctly as powerful an agent in preventing the changes which take place in paralyzed limbs in the disease we are considering, as that which electricity seems to afford us? Or may we go further, and ask whether, by artificial movement and heat combined, we do not possess all that is required for the sufficient treatment of this class of cases?

If we can assure parents that, by attention to those details which common sense will suggest in the direction indicated, and desire particularly that the child shall be brought, at intervals of three or six months, for the purpose of comparing present with previous conditions, insisting much on careful daily attention to instructions given—I say, if this plan be pursued, will not the results be altogether more satisfactory than the plan now adopted of sending a child to the hospital to be galvanized or faradized once or twice a week, or, failing this, to leave it to the probable fate of some quack treatment more injurious than total neglect?

On some future occasion I shall give fuller reasons for thinking that artificial heat is the most powerful agent we have in preventing the wasting of tissues, which is the striking feature in this form of paralysis.—*Robert J, Lee, M.D., in British Medical Journal.*

---

The Best Methods of Treating Operative Wounds—A wound *per se* is not benefited by carbolic or other medication. No student of Mr. Lister would for a moment claim that a wound is other than irritated thereby and rendered less likely to quick repair, but, that it is treated in this manner as the le.s of two evils, for thus we exclude the contamination of putrefactive agencies, and secure an aseptic wound, surgically clean in character, as nearly as possible like a subcutaneous injury.

Do facts sustain the extraordinary claims of antiseptic surgery? It would seem that a verdict could be obtained from such a tribunal, from which there could be no appeal· and thus settle the question at issue. Yet in such a problem, it is very difficult to obtain reliable data, simple as it would at first appear. Facts seem to have an especia. aversion to registration. Percentages of death rate fall far short of a representation of the care. Amount of suffering and pain, depreciation by suppuration and exhaustion, the length of the period of retention under surgical observation, and detention from active employment; all these factors come within the scope of experience. The lesson to be taught from statistical research is the proud record of recent, sudden and marvellous improvement, and whether under antiseptic regime or not, by far the most important factor appears in the scrupulous attention to cleanliness, as a "virtue next to godliness," and this, when perfectly attained, by whatever means, is no more or less than an aseptic wound, the theoretic as well as practical result sought and secured by the most careful devotion to antiseptic detail. Much of statistical evidence has been adduced which needs no repetition here.

It is scarcely more than a decade, when the mortality rates were so great in Boston and vicinity, after ovariotomy, that such leaders of the profession as Drs. Bigelow and Wyman, denounced the operation as unwarrantable. and decried the men who presumed to think differently. Now how changed!

Ovariotomy is not looked upon as a very dangerous operation, and this is due in very large measure to the observance of the great principles of antiseptic surgery.

Yet it is just here, in the field of abdominal surgery, Mr. Lister would regard his innovations as the least triumphant.

Sir Spencer Wells' marvellous experience stretches over a wider range than any other ovariotomist, and he gives it as his unqualified opinion that the greatest gain both in safety and rapid recovery is due to these protective agencies. We have elsewhere referred to Dr. Keith, who in abandoning the spray for especial reasons, by no means ignores or fails to put them in practice. Indeed, Dr Keith himself reports eighty successive recoveries while using the spray, a record without duplication in the history of surgery.

Mr. Thornton believes that almost the only danger in abdominal surgery is septicism. He reported last September at the Boston Meeting of the American Gynæcological Society, that of his last ninety cases of ovariotomy he had only three deaths, and these three were malignant cases. Under such protective influences, uterine tumors are removed, and the entire organ with the ovaries is ablated with fair results.

Did the occasion permit, we would gladly review the entire realm of operative surgery. The broad clinical fact pertains to the entire field. Blood poisoning, arising from altered secretions in the wound, which has hitherto been the ever present dread and danger, is prevented. If this is owing to putrefaction which depends upon the admission to the wound of the minute living particles, and if, by the careful application of certain precautions, these changes and their consequences may be with certainty prevented, then all these various forms of septic and pyæmic processes may and should be eradicated.

The treatment of compound fractures and the open wounds of the larger articulations to my mind offer the most astounding proof of the great value of antiseptic precautions.

While admitting that a depraved constitution and a devitalized condition would render the patient less able to resist the depredations of these vital poisons, he asserts as a demonstrated clinical fact, that such patients have a revivifying bio-plastic power sufficient under all circumstances to afford immediate and complete repair if treated antiseptically. Drainage is even more important in antiseptic treatment than without. Under the irritation of the germicide used, there is an increased exudation from the divided portions, and unless this be removed there may be tension and separation of the parts. *Disinfection, close approximation, drainage, rest* and *protection* are the vital factors in modern operative wound treatment.

The wide experience of the profession with carbolic acid as the germicide most relied upon, has given rise to a series of dangerous and fatal issues and causes it to be held in fear or disuse by very many who recognize its antiseptic value. This has led to experimentation with other agents, a very considerable number of which are recommended and sold for this purpose. Careful laboratory experiments have been undertaken by my assistant, Dr. Samuel Nelson, and myself in order to test the relative values of a number of antiseptics. For the destruction of organisms which cause putrefaction in wounds the agent to be selected must act quickly.

From these experiments necessarily imperfect, although occupying a considerable period of time and attention, we may deduce that carbolic acid still holds its place as the best agent for the rapid destruction of micro organisms and that thymol, salicylic acid and the fluid called Listerine prepared by Lambert & Co., of St. Louis, are germicidis of a trustworthy character. Any agent which is as safe as the latter and as promptly active as the former would be a valuable addition to the surgeon's armentariun—*Henry O. Marcy, M.D., in Med. Gazette.*

---

STYPTICS IN GENERAL SURGERY —If hemorrhage is sufficient to make its arrest by surgical means important, styptics are either worthless because inefficient, or needless because better hæmostatic measures are easily applicable. That which is inefficient and unnecessary is certainly useless. Hence styptics are useless for arresting hemorrhages met in general surgical practice.

By styptics I mean those astringent chemical agents that are employed to stop bleeding, because of their tendency to produce contraction of the vessels and surrounding tissues, and because of their effect in inducing rapid coagulation of blood. Their number is great. Subsulphate of iron, perchloride of iron, alum, tannic acid, gallic acid, turpentine, the copper, zinc, and silver salts, and combinations of various mineral and vegetable ingredients, have had their advocates. They are all about equally useless, though some are more objectionable than others.

The method of using styptics generally recommended is substantially as follows: "Remove loose clots, wipe the bleeding surface dry, and press upon the part a piece of cotton, muslin, or sponge impregnated with the styptic powder or solution." In many cases this will, I admit, be followed by cessation of bleeding; but so would mere exposure to the air, or the application of pressure without the styptic solution.

I have three objections to the use of styptics:

1. Their reputation as hæmostatic agents leads practitioners to resort to them when more trustworthy methods are needed. Thus valuable time is lost, for, after temporary arrest, the hemorrhage recurs in the already anæmic patient, and is perhaps followed by disastrous results.

2. If they fail to control the bleeding—which they generally do if the hemorrhage is important—it is often so difficult to rid the surface of the pasty clots that subsequent ligation of the vessels is wellnigh impracticable.

3. Many styptics prevent union by first intention, because they irritate the raw surface, lead to inflammation, or induce suppuration.

Monsel's salt—the subsulphate of iron—has probably more reputation than any other styptic, yet it is the most objectionable of all. It covers the wound with black, sticky clots, which obscure further examination of the surface, prevent primary union, and may even allow bleeding to occur beneath them. I have seen such leathery masses of coagulum raised up into vesicles by the subjacent hemorrhage.

There are but two scientific and satisfactory ways of arresting hemorrhage as usually observed in the practice of general surgery:

1. The first is occlusion of each individual vessel by ligation, torsion, or acupressure, and is generally not required for arteries smaller than the facial, nor for veins, except those of the largest calibre.

2. The second method is direct pressure by compresses and bandages, which, if properly applied, will always be effectual when the first method is not demanded. It is to be adopted when there is oozing from small arteries and capillaries.

In all cases of traumatic hemorrhage, it should be recollected that a man can lose many fluidounces of blood without serious injury, and also that no artery or vein can bleed if it is compressed by the fingers. These facts assure the surgeon that there are always time and means to control the bleeding at least temporarily.

Many arteries that spurt freely when first divided soon spontaneously stop bleeding. Therefore it is foolish to interrupt the steps of an operation by ligating every little vessel that throws out a jet of blood. Let the surgeon proceed, even if the arteries are quite large, and when he has finished his incisions he will find, to his surprise, very few points requiring ligatures. He should ligate these, and after washing away the loose clots, make moderate and equable pressure. There will then be no part for styptics to play.

It is possible, perhaps, that there may be occasional instances of oozing where pressure cannot be effectually applied; but these are certainly so rare that they do not materially affect the truth of the proposition that styptics are useless. In bleeding from cavities, compressed sponge will often make efficient pressure; and with elastic bandages we can obtain sufficiently firm compression even of soft and flaccid parts. Of course bandages must not be applied tightly enough to strangulate and cause gangrene. Firm pressure is all that is necessary, for it requires only moderate digital pressure to occlude even the largest arterial trunk.

It would be well if the profession could be made to forget the very existence of styptics, for then every one would treat hemorrhage by the best methods, and the waters of Pagliari, Ruspini, and Brocchieri would deservedly cease to flow, and would soon sink as far from sight as their ancient inventors are buried. When the physician again treats ague with the bark jacket it will be time enough for the surgeon to treat hemorrhage with styptics.—*John B. Roberts, M.D., in Philadelphia Med. Times.*

---

Suppurating Synovitis of the Knee—The knee-joint, like other portions of the body, is liable to concussions; the effect of forcible contact of their surfaces, as in indirect or transmitted violence. Direct violence may likewise produce but little injury to the surface, the force of the blow exploding, as it were, in the joint. The effect of such an injury is manifested primarily by the synovial membrane, like other serous membranes, has the peculiarity of secreting a large quantity of serum when inflamed. As a consequence of this we have swelling, at the same time the membrane loses its lustre and smoothness, and in the process of the inflammation white corpuscles escape from the vessels and ultimately degenerate into cells. Thus the fluid in the joint is constantly becoming opaque and purulent till finally it has all the characteristics of pus. Such is the condition of this man's knee, the effusion which at first consisted merely of synovial fluid has degenerated into pus, which matter in accordance with the law of nature, in its endeavor to escape, has burrowed its way out into the structures covering the joint, so that here upon the outer side we have an abscess pointing, which abscess, no doubt, communicates directly with the cavity of the joint. The tension caused by the accumulation of the pus in the joint has caused an irritative fever, of a hectic character; hence the source of constitutional symptoms above referred to.

Should the proper treatment not be instituted, the abscess would open, discharge its contents, and probably the patient might recover with an ankylosed knee. It is more likely the entrance of air into the joint would lead to putrefaction, which would result in total disorganization of the joint, or to death of the patient. In any event the prognosis of the case is a grave one. Should we save the life of the patient, the functions of the joint may be more or less impaired, for we cannot say with certainty how far the disease has extended, it may involve the cartilage, or even the bones; be that as it may, the line of treatment is clearly indicated. We shall anticipate nature's work, and by free incision into the joint let out the pus. The patient anæsthetized, we make an incision first into the abscess, and several ounces of pus escape. Now should we treat this as an ordinary abscess the wound would heal, and as we have pus constantly secreted from the diseased synovial membrane, it would soon reaccumulate, and we would have a return of this condition. In order to prevent such an occurrence, I shall make a counter opening, which shall likewise extend into the joint. Now, in order to facilitate drainage and allow the matter to pass off as quickly as it is formed, I shall pass into the cavity of the joint a drainage tube, the ends of which shall project at either opening. Through this tube we throw gently a stream of water, pure or carbolized, thus removing all inflammatory products from off the synovial membrane. We direct this irrigation of the joint to be repeated every few days, so long as pus is discharged through the tube. When the discharge lessens materially this drainage tube shall be withdrawn and in its place two short ones introduced at either wound, these in turn are to be gradually withdrawn as the discharge ceases.

But this is not all, the most important, feature of treatment, in this as in all inflammations, is rest to the part. This is best afforded in this particular case by the application of a well adapted plaster of Paris splint. This splint should extend from the middle of the thigh to several inches below the knee joint. The object of this splint, is to hold the part absolutely at rest. With this apparatus applied, and the tube *in situ*, we place the part in the most favorable condition to recovery. In addition to this local treatment, we shall endeavor, with constitutional remedies and good hygiene, to restore his lost vigor.—*W. T. Briggs, M.D., in Nashville Journal of Med. and Surg.*

---

TREATMENT OF PARTIAL TRICHIASIS BY ELECTROLYSIS.—To the negative electrode of a Leclanche's battery, I attached a rather fine gold electrolysis-needle, and inserted the point of this to a depth of about four or five millimetres along the hair to be destroyed, so that its point should reach well above the root. I then applied the positive electrode to the lid near the outer canthus (having first made sure that the battery was working sufficiently well); contact was conveniently made by wrapping some wet cotton wool round the end of the wire. In a few seconds, the tissues immediately around the needle (negative electrode) began to show white, and soon a distinct bubbling of hydrogen gas could be observed. Half a minute or so, according to the strength of the battery, usually sufficed to completely loosen the hair; I then withdrew the needle and the positive electrode, and, with the fingers or a forceps, removed the hair. It should come away without requiring the slightest drag, and bring with it a gelatinous-looking mass of dead tissue. If the hair be not sufficiently loose, the needle must be reapplied for a few seconds; but, with a little practice, the required time can usually be guessed with accuracy. Each hair has, in this manner, to be destroyed.

The amount of inflammation of the lid resulting is usually not great. In one of the first cases in which I tried electrolysis, an abscess formed, owing, no doubt, to my having inserted the needle too deeply; but that was the one and only untoward result which I have seen in about 120 hairs so treated.

Four of Leclanche cells are usually enough, if the battery is in good order; but the current must be strong enough to decompose water with facility.

It seems not to be advisable to destroy very many hairs in the same lid at a sitting, for the resulting swelling is sometimes considerable. The advantages which I claim for this method over epilation, snaring, or the actual or potential cauteries, are these:

1. Any individual hair can be destroyed without injuring those beside it.

2. The hair can be got rid of at once and forever.

3. Hairs of any length, strength or position can be treated.

4. By its use, it will render unnecessary many of the more formidable operations on the lids, besides saving the patient much misery.

In cases where a very large number of hairs are misplaced, or where entropium exists, it is inapplicable, and some of the numerous plastic operations should be preferred.

The greatest disadvantage, as far as I have seen, is that it is very painful for the time.

I have now been practicing this method for over four months, at St. Mark's Ophthalmic Hospital, during which time I treated 120 ciliæ by electrolysis, and had some of the patients under constant observation for a month, and at intervals since, and have been able to observe no recurrences; and have been so pleased with the results obtained, that I have ventured to bring the subject before the ophthalmological section of this association, feeling sure that to those who, like us in Ireland, have a large number of cases of trichiasis to treat, it will prove a valuable help, and that by its means many an one who had been for months a regular attendant for epilation to the *clinique* may be permanently cured.

Four months may seem too short a time to have tested the permanency of the cure; but, while freely admitting this, I must urge that by no other method that I am acquainted with have hairs been for so long kept from showing; and, from the nature of the case, there is every reason to expect that the vitality of the follicles have been quite destroyed, and consequently no return is possible.

If applicable for the ciliæ, electrolysis should be equally applicable for the destruction of hair-follicles elsewhere, in moles, or hairy-faced females.

The method can, however, only be regarded as on its trial; but, so far, the results have been such as to encourage the most sangnine hopes, and to invite a much more extended trial.—*Arthur Benson, M.B., Dublin, in British Medical Journal.*

---

URTICARIA—The exciting causes of urticaria consist in certain forms of irritation of the terminal nerves, acting either within or without the body. How these irritants act is a question not always easy to answer, but that the relation which they bear to the eruption is one of cause and effect is often evident to the most careless observer.

It is not only by reflected nervous irritation that gastro-intestinal derangement evokes urticaria, but in a larger number of cases the imperfect digestion of the food undoubtedly induces a state of the blood which tends to excite irritation in the skin itself. We know that a certain systemic condition, not always easily demonstrated, is the very essence of gout and rheumatism. The same or a similar condition is regarded by most dermatologists as being at the root of eczema and psoriasis, and there is no less reason to assume that this is a potent factor in the etiology of urticaria. Certainly, the beneficial effect which so often results from a judicious stimulation of the liver and kidneys would seem to justify the assumption.

Uterine and menstrual derangements constitute a prolific source of certain phases of cutaneous disease, and to this source certain cases of urticaria may possibly be referred. The eruption has been known to occur in successive pregnancies, and its occurence after the application of leeches to the os uteri has already been mentioned.

Many other pathological conditions might be cited as possible, if not probable, causes of urticaria. Still there would remain one important factor to consider, viz., idiosyncrasy. It is impossible to explain this "mystery of individuality." We can no more say why only one person in a thousand, or thereabouts, should suffer from urticaria after eating lobster than we can say why one among many should escape after thorough exposure to an infectious disease. Not one of the internal causes of urticaria which have been mentioned will alone suffice to produce the eruption. There must be a certain sympathy existing between the skin and the internal organs, a peculiar temper of the nerves which is born with the individual, and which cannot be regarded as a pathological condition. In the production of urticaria, a most important part is played by the sympathetic, and particularly the vaso-motor nerves. The same remark, however, will apply with equal truth to many other affections of the skin, and there is no reason, therefore, regarding urticaria as being in any special sense a neurotic disease.—*Geo. Henry Fox, M.D., in Jour. of Cutaneous and Venereal Diseases.*

---

Treatment of Icterus Neonatorum.—I must begin my remarks on the treatment of congenital jaundice by saying a few words regarding the treatment of the spurious kind, which, in order to make its title accord with its pathology, I have changed the name to "chlorsis neonatorum." From having said it is a morbid state met with in immature and weakly children, it can be readily understood why not medicine but good mother's milk, external heat, and plenty of fresh, dry, warm air must be the essentials of treatment. When I have said this, I have said all that is necessary to be said upon the subject. So now I turn to the consideration of true congenital jaundice, for the one form of which we can do much, for the other nothing at all.

The treatment of congenital jaundice from suppression of the biliary function is in most cases a very simple affair. A single dose of a mercurial being in general sufficient to dispel it. But here I have an important word of advice to give, and that is, to advise my readers *not* to follow the plan recommended in the books of giving either the gray powder or the calomel to the baby ; on the contrary, give the medicine to its wet-nurse, whoever she may be, for a dose of mercurial given to the woman who suckles is sure to affect the child who sucks her ; aye, and that, too, much more naturally and satisfactorily than if the medicine was poured down the infant's own throat. There is no fear of the mercurial doing the mother harm, even if it be given within seventy-two hours after confinement, as I well know from personal experience, or I should not thus unhesitatingly recommend it.

I need say little about treatment in cases of jaundice from a congenital deficiency of the bile ducts for, of course, they must inevitably sooner or later end fatally.

One might expect, even after what was said in the physiological chapter, on how very essential to life is the presence of bile in the digestive process, that it would be almost impossible for a child to survive beyond a few days or at the very utmost beyond a few weeks, with an entire absence of any canal by which the bile could obtain access to the duodenum, yet, strange to say, Dr. Nunneley has put on record the extraordinary case of a boy, who was born jaundiced in consequence of a mal-formation of the bile duct preventing bile from reaching the intestines, and actually lived for nearly seven months. At the time of his death he was deeply jaundiced and emaciated. Emaciated naturally enough he would be from no bile having reached the chyme to prepare and fit it for the process of intestinal absorption and assimilation.

Even in the apparently most hopeless cases, as it is just possible that an error may be made in the diagnosis —for infantile affections of this kind are not easily differentiated—it is always advisable in the first instance to try the effect of a mercurial, given, as above said, through the instrumentality of the wet nurse.—*George R. Harley, M.D., F.R.S., in Diseases of the Liver.*

---

QUACKERY IN AMERICA.—Accustomed as we are to consider the States as the Elysium of quacks, the records of the doings of these pretenders, published from time to time, arouse a feeling of increased astonishment at each revelation. Beplagued by its army of sham practitioners, and abundantly spotted with villainous institutions whose founders drive a profitable trade by selling bogus diplomas, the United States may well be considered to deserve the sympathy of all who are able to appreciate the frightful mischief annually done to the population by uncontrolled practitioners of every conceivable shade of humbug. In Missouri, which the New York *Medical Record* describes as "a quack-ridden State," (what about New York itself?) there are 4,834 practitioners of one kind and another, one doctor to every 450 inhabitants. Of these, 3,453 "belong to regular medicine," "and over 1,300 are eclectics, homœopaths and nondescripts. But hardly more than one-half (2,456) are *graduates* of regular schools." In addition to these facts, which are quoted from a paper of Dr. King, President of the Missouri State Medical Society, the author of the communication referred to, declares that 269 of the practitioners are abortionists, 1,904 are deemed by competent judges to be incapable of practicing medicine intelligently, and 452 are persons of immoral character, 34 being women. The accounts of the damage perpetrated by this army of rogues, however, is most instructive. It includes 5,570 lives annually lost through quackery in the State, in addition to 8,000 children killed *in utero* by the 269 abortionists; the yearly sum paid for these services reaches somewhere about half a million sterling; verily, a pleasing reflection for those who love their species! Some of the instances of cure performed or promised by quacks are interesting from a professional point of view. They are, many of them, curious in the extreme; occasionally they are unique, as, for instance, the promise of a "medical man" who undertakes to cure nasal catarrh at one sitting, and, in proof of performance, "remove the catarrh and place it on a saucer." English quackery has not yet reached the perfection of American samples. Long may it continue to hold a position of inferiority.—*Med. Press and Circular.*

Removal of a Vesical Calculus Weighing Three Thousand Five Hundred and Forty-One Grains From a Boy Sixteen Years of Age.—There are many points of interest connected with the following case of urinary calculus. It is the largest calculus ever found in the bladder of a patient at so early an age as sixteen.

The history of the case is as follows: Patrick Colbert, aged sixteen, residing at Greenpoint, L. I., presented himself at my college clinic, in Bellevue Hospital Medical College, on October 23d. He complained of pain in the region of the bladder during the act of urination, pain in the loins, and on the inner side of the thighs. There was a frequent desire to micturate, and a great increase in the pain during, and also after, the act. He first noticed pain during urination seven years before, but gave little or no attention for a couple of years, until he noticed a great deal of sediment in the urine. He then consulted a physician in a city dispensary, who gave him medicine for what he called the gravel. The treatment relieved him for a while, but the painful symptoms returned again with renewed violence. Notwithstanding his illness, he was able to work at his trade in a junk-shop; but within the past twelve months he had frequent attacks of pain, accompanied with fever, compelling him to go to bed, where he would remain for several days, and then again resume work. An examination with a sound revealed the presence of a large stone. With the finger in the rectum its unusual dimensions were easily ascertained. Notwithstanding this, I deemed it advisable to attempt its extraction through the perinæum, thinking that I might be able to crush it with a lithotrite. I was assisted during the operation by Professor Dennis, and by Drs. Schweig, Waldo, and Willis.

The patient was etherized, and a grooved staff was introdued and held in position by Professor Dennis. An incision was then made in the median line, an inch and a half in length, down to the urethra, which latter was opened almost to the same extent. Wood's bisector was then passed on the grooved staff into the bladder. The opening thus made admitted the index finger with great ease, when the size and general character of the stone were readily defined. Although convinced that it would be impossible to crush it or remove it through the perinæum, I passed in Civiale's lithotrite, and endeavored to grasp the stone. Failing with this, I then tried Bigelow's instrument with a like result. Ceasing all efforts in this direction, the patient's position was changed and an incision three inches in length was made in the median line above the pubes, and continued down to the bladder. A sound introduced through the perineal wound into the bladder facilitated the incision through the anterior wall of the viscus, which was made from the border of the peritoneal fold above to the pubes below, fully exposing the stone. The bladder was then found to be firmly contracted on the stone in such a manner as to render it impossible to enter a forceps on its sides sufficiently far to grasp and extract it. After several vain attempts to remove it, both with the fingers and with the forceps, I passed my right index finger into the bladder through the perineal opening until it impinged on the lower border of the calculus, while above I grasped the rough projecting part with the fingers of my left hand. Then, by pushing with great force upward with the index finger and lifting with my left hand, I was enabled to force the calculus out of the bladder. It was held so firmly that several minutes were consumed in this manœuvre alone. During the manipulations through the abdominal incision the peritonæum was opened, and a loop of intestine came down. This was returned to, and retained in, its natural position without difficulty. The stone measured three inches in its longest diameter and two and a quarter inches in its transverse diameter. Its weight, as ascertained by Mr. Peabody, at Caswell, Hazard & Co.'s, was three thousand five hundred and forty-one grains.

After the removal of the stone, a piece of ice was placed in the bladder, to control the hæmorrhage which took place from a spot on the floor of the organ, to which the stone seemed to have been attached. As there appeared to be free drainage through the perinæum, I concluded to follow the old rule, and not place any suture in the walls of the bladder. The opening in the walls of the abdomen was closed with silk sutures, excepting at its most depending portion, where a space of half an inch was left for additional drainage.

The patient was ordered a teaspoonful of the United States solution of morphine, to be repeated every two or three hours until the respirations were down to thirteen a minute. In all operations where the peritoneal cavity has been opened, designedly or by accident, I have been in the habit of administering almost as much morphine as if the patient already had peritoneal inflammation. I am inclined to the opinion that peritonitis may often be averted by this treatment.

The patient recovered from the effects of the ether in a short time, but about an hour afterward began to show signs of collapse. His extremities became cold, and his pulse weak and fluttering. The house surgeon, Dr. Seabury, administered stimulants freely, and he soon rallied, seeming to be very little the worse for the operation. On the morning following, his temperature rose to 103° Fahr., and he complained of pain in his back, head and limbs. The urine was oozing freely through the lower portion of the abdominal wound, and through the perinæum. On the third day after the operation (Wednesday), the urine had ceased to flow through the perineal wound, owing to the inflammatory tumefaction there, but it escaped very freely through the lower part of the opening in the abdomen. A small quantity also came away through the urethra. In order to make the drainage perfect, another suture was removed in front, which left over an inch of the incision at its lower part open. This was necessary, in order to insure the absolute safety of the peritoneal cavity, which had been opened during the operation. His temperature at this time was 102° Fahr., pulse 98. A gum-elastic catheter was introduced through the perinæum into the bladder, and allowed to remain. The following day, as only an ounce of urine had passed through the instrument, and as it occasioned considerable pain, it was removed, leaving the drainage to the abdominal opening. The urine flowed constantly at this point, and a few drops also escaped through the urethra. The temperature was down to 100° F., and his pulse ranged between 90 and 100. On Friday, the urine began to have an ammoniacal odor, and he complained of some pain in the hypogastric region. The bladder was then washed out with carbolized water, and afterward with a solution of borax. These washings were repeated three or four times daily until the opening got too small for the catheter.

Four weeks from the date of the operation the abdominal wound had entirely healed, and all the water was passed by the natural channel. He left the hospital a few days later, entirely well.

From a careful study of this case, and from an examination of the records of the extraction of large calculi through the perinæum and rectum, I am convinced that the supra-pubic operation is the only safe one. The stone can be removed without lacerating important organs. Free drainage can be kept up through a perineal opening, as well as through the lower extremity of the abdominal incision, thus reducing to a minimum the danger arising from urinary infiltration and peritonitis.—*Joseph W. Howe, M.D., in New York Med. Journal.*

---

TONSILLOTOMY.—In the medical literature of the day, and in conversation with individual members of the profession, I find the greatest difference of opinion as to the wisest treatment of hypertrophied tonsils and the best method of its application.

First of all, by excision we do not now mean the complete extirpation of the tonsil, but rather the removal of so much of the hypertrophied mass as protrudes beyond the muscular folds. It will be remembered that the hypertrophy is a true hyperplasia, an increase and induration of the connective tissue; that the follicles are enlarged, and the walls of the ducts thickened and sometimes distended with retained secretions, and that the epithelial covering is also thickened.

When the tonsils are thus changed, repeated attacks of acute inflammation often occur, which alone would suggest interference and permanent reduction, but this is by no means the most weighty reason.

The existence of an obstruction at the very gateway of the respiratory tract cannot but do harm. When the tonsils enlarge toward the median line the air current is necessarily interfered with, especially during sleep, when the relaxation of the muscular surroundings favors a nearer approach to each other of the hypertrophied masses; what wonder then, the supply of air being limited, that the child becomes pale and weak, with poor assimilation of food and diminished power of resistance to disease. In many such cases a marked improvement in general health and appearance begins soon after removal of the enlarged tonsils, without any other treatment.

Again, from the narrowing of the air passage and the consequent labored breathing, the auxiliary muscles of respiration and the diaphragm are called into action to a greater extent than usual, and deformity of the chest is often the result.

Still further; it is reasonable to believe that damage may accrue to the lungs and bronchial tubes from this increased respiratory effort. The air being drawn into the lungs with difficulty, a rarefaction to some extent at once occurs within the tubes and vesicles, and a consequent dilation of the capillaries of the bronchial mucous membrane and intra-alveolar walls. Where the obstruction was great I have not infrequently found bronchitis, which speedily yielded after removal of the tonsils.

If the tonsils be large enough to obstruct expiration also, a prolonged expiratory murmur at the pulmonary apices is sometimes heard.

That hypertrophied tonsils may cause and keep up pharyngeal irritation is so plain a proposition that it is needless to argue it. On the other hand, it is not likely that Eustachian obstruction is ever the direct result of tonsillar hypertrophy, though it may be caused by the extension of the pharyngitis from such a source.

Enough has been said to suggest something more than expectant treatment where the tonsils are chronically enlarged to such an extent as to interfere with the free passage of air, or cause pharyngeal irritation.

There are several methods of treatment commended, each having earnest advocates Some physicians urge general tonic and nutritive medication, believing that the system may be encouraged to resist the consequences of hypertrophy and to cause its absorption. This is well as far as it goes, but it does not remove the cause, nor is it alone to any extent successful.

The use of sprays and the application of remedies with a view to produce absorption have long ago been proven insufficient--as well spray a fatty tumor or a sebaceous cyst—while the practice of trying to reduce pharyngeal inflammation before the tonsil is removed is in inverse order.

The results following the use of chromic acid, the London paste, and even the electrolytic needle, have not been satisfactory. It is true that Cohen has had success in a few cases following 15 or 20 operations with the galvano-caustic, and it is also true that repeated local applications may do something toward reducing soft and yielding tonsils, but where there is true hypertrophy, large, firm and tense, these are of little account, except that they prolong treatment and continue the case. Wagner says: "That after patient, persistent and regular applications continued through months, he has never seen a true diminution in size."

Where there is true hypertrophy once, and any considerable degree of obstruction, the most rational—I had almost said the only rational—treatment is excision. In former days this was done with the bistoury and forceps, a somewhat painful and difficult operation. The introduction of the guillotine has done away with many of the objections once urged. I use a simple instrument, having but two pieces, a chisel-shaped knife, sliding, when in position from before, backwards in the groove of a guard ring, and closed by a single motion of the thumb. Only what passes through the ring of the guillotine is severed; there is no danger of wounding the tongue or pharynx, and the section can be made in an exact plane with the level of the pharyngeal pillars. The muscular folds, if adherent to the tonsil, should be detached before the operation.

A few of the objections to the operation will bear notice. Some fear the pain will be great, but the hypertrophied tonsil is not sensitive; even little children seldom complain of it, and with the guillotine at most it is but momentary.

Hemorrhage is rarely met with and seldom lasts long. Guersant and Voss, out of 1347 cases, had but three in which there was any considerable bleeding, and these were probably due to the tonsil being pulled out of position by forceps, and a deeper section made by the bistoury than needed. It is probably best not to use even a guillotine, which is so constructed as to lift the tonsil from the pharyngeal wall before section. I have yet to find recorded a single case of alarming hemorrhage following the use of a properly made and applied instrument. Where there is any tendency to bleeding, after the first two or three minutes, it can be easily checked by the tannic and gallic acid gargle, recommended by Mackenzie. Even this I have never had occasion to use.

The proposition advanced that removal of the tonsils destroyed virility in the male, did not survive investigation.

Without recapitulation, let me add that I have never regretted doing the operation, but in some cases regretted that it was not done.—*William Porter, M.D., in the Weekly Medical Review.*

---

Pop-Corn for the Vomiting of Pregnancy.—For the past four years I have used for this complaint a simple remedy, which has uniformly given me great satisfaction, having *always* found it a *beneficial* and *generally* a *sufficient* remedy. It is the quickly-roasted grain of a species of Indian corn, too well known as pop-corn to need any description. It should be popped in a wire popper, not (as is sometimes done) in a spider with grease; should be white and light, sprinkled with a little salt, and eaten freely. I might state many cases in which this simple remedy has "acted like a charm" in this disorder, but will content myself with the relation of the following one:

On the 8th of January I was called to visit a lady advanced a little over three months in her first pregnancy, who had suffered from almost constant nausea and uncontrollable vomiting from the time of her conception. I found her in bed, to which she had been confined for more than four weeks, pale, languid, depressed in spirits, pulse 120, feeble, and so prostrated as to be unable to raise her head from the pillow. For the past sixteen days her stomach had absolutely refused to retain anything; everything—food, medicine, even a swallow of water, being instantly rejected. During the past six days, absolutely nothing had been taken into her stomach; all nourishment and medicines having been administered per rectum. Even then she had constant nausea, almost continuous violent retching, and frequent vomiting of bilious matter. For a long time she had been under the care of a respectable practitioner, who had faithfully tried nearly all the usual remedies (?) for this complaint. Fowler's solution, oxalate of cerium, bismuth, ingluvin, "*et id omne genus*," had been given internally. Copeman's method of dilatation, nitrate of silver in substance and in saturated solution, had been applied to the os and cervix. Sinapisms, hypodermics of morphia, ether spray to the epigastrium and spine, injections of bromide potass. in beef tea, and I cannot remember what else, had all been faithfully and unavailingly used. Some pop-corn was immediately prepared, a little salt sprinkled thereon, and of which she ate a large saucerful. At my call next day, found she had passed a quiet night, without nausea or vomiting, and had eaten very freely of the pop-corn. She has had no further trouble. January 25, as I was passing her house, I saw her at the door. She informed me that since my first visit she has vomited but once, and then but slightly. Sometimes has slight nausea, which is always relieved by eating some pop-corn.

The remedy is so simple, so easily procurable, so utterly harmless, and with me has always proved so efficacious, that I publish this in the *Reporter*, confident that my professional brethren will find it singularly efficacious in the relief of this most distressing disorder.—*T. C. Wallace, M.D., in Med. and Surg. Reporter.*

Syphilis of the Spinal Cord.—D. F. Greiff, Heidelberg. *Arch. f. Psych. und N.*, xii, 3. The author calls attention to the imperfection of our knowledge on this subject, compared with that of cerebral syphilis. The anatomical changes found in the latter consist of specific neoplasms, either as circumscribed gummata, or diffused gummatous infiltrations; specific inflammatory processes of the meninges and adjacent cerebral substance; and, finally, syphilitic disease of the cerebral arteries and its effects, first described by Heubner. Reviewing the literature of syphilis of the cord, he finds: (1) cases of circumscribed gummata, in some originating from the envelopes of the cord, and in others developing within the cord itself (cases of Rosenthal, MacDowell, Willis, Wagner, Hales). (2) Diffused neoplasms (cases of Zambaco, Bruberger, Westphall, Aeubner). (3) Inflammatory changes in the meninges associated with further changes in the cord itself (cases of Homolle, Winge, Charcot, and Gombault, Schultze, Julliard). No description is to be found of specific diseases of the vessels of the spinal cord. The dilated vessels, with thickened walls, surrounded with cellular infiltration, described in some cases, as they appear under other circumstances also, cannot be acknowledged as specific. The same doubt must remain in all casses where inflammatory processes of the cord and membranes are unaccompanied by specific gummatous tissue, and notwithstanding many attempts, the problem of determining the characteristic anatomical relations of syphilitic myelitis remains, he thinks, unsolved. He gives an abstract of 13 cases with autopsies, and refers in detail to Juillard's views, who considers that the combined presence of inflammatory processes in the meninges, of exudative processes in the vessels and their sheaths, and then hyperplasia of the neuroglia with its effects upon the neural elements, constitute the characteristics of syphilis of the spinal cord. As the pathological processes involve principally the lymphatic system of the cord, entering through the meninges, the neuroglia, and the vascular sheaths, it results that the changes may be diffuse, but they cannot constitute a "systematic" disease. If the process be a rapid one, softening follows; if slow, sclerosis. The author reports very fully his own case, in which the changes consisted of an extensive inflammation of the pia—in some portions just beginning, in others at an advanced stage; decided disease of the arteries and veins, also a swelling and hyperlasia of the interstitial tissue, with inflammatory exudations around the vessels, and moderate involvement of the neural elements. The arterial changes were the same as those described by Huebner for the brain, observed in the cord for the first time. A peculiar obliteration of the veins also existed. No softening or decided "systematic" lesion was found. He concludes that the facts of this case support Juillard's views, and furnish undoubted proof of the existence of a syphilitic disease of the arteries of the cord, but while the changes in the meninges and the vessels appear to be undoubtedly of a specific character, the changes in the cord, on the contrary, exist only in connection with and dependent upon the former, which together represent the true character of syphilis of the spinal cord.—*Journal of Nervous and Mental Disease.*

---

The Dangers of Amateur Doctoring.—A painful illustration of the dangers that may be associated with amateur doctoring has recently been afforded by the death of a girl at West Malling, in Kent, through poisoning by oil of almonds, administered by the Rev. J. H. Timins, rector of the parish. The deceased, a young girl, daughter of a laborer, was a parishioner of the clergyman in question, who seems to have largely interested himself in the material as well as the spiritual ills of his flock; and in pursuance, no doubt, of very worthy desires, constituted himself their medical attendant on frequent occasions. The girl whose death he now stands charged with causing was seen by him to be in a state of bad health, and as an agreeable remedy under the circumstances he prescribed and administered to her a considerable dose of oil of bitter almonds obtained from a local chemist, by whom, however, he was duly warned as to its poisonous properties. The defence of the reverend gentleman was based upon the fact that thirty years ago he studied medicine for a time at St. Thomas's Hospital, and that he treated the patient for apoplexy, from which he judged her to be suffering; also that oil of almonds was a harmless remedy previously used by him in the case of his own son, and so far innocuous that he had himself taken a spoonful from the same bottle out of which the dead girl had received it. It is a pitiful necessity that we are under which compels us to comment on this case at all. There can be no question that the reverend gentleman was actuated by the most benevolent intentions in the matter; there can equally be no question that the indiscriminate adoption of such principles of action by clergymen, or, indeed, by any non-professional person, is fraught with extremest danger, even to life itself. In the present instance a coroner's jury has been compelled to register a verdict of manslaughter against the Rev. Mr. Timins, who consequently stands committed for trial on that count. There is, perhaps, more of injustice than of right in a condition of things which leaves it possible for such an unpleasant complication of social relations as this to be brought into existence; and until the time arrives when it shall become a criminal matter for any unauthorized person, in the absence of dire emergency, to take upon himself the office of a medical practitioner, it can never be that human life will be safe from the pretensions of ignorant busybodies or the yet more dangerous trading of quacks. Mr. Timins is unable to urge even ordinary necessity as an excuse for his interference; medical men were resident within immediate vicinity of the house in which the death occurred, by summoning whom it is open to us to believe the catastrophe might have been avoided.—*Med. Press and Circular.*

---

Artificial Illustrations of Percussion Sounds.—Dr. Austin Flint, in the course of an interesting series of lectures delivered before the Philadelphia County Medical Society on "The Physical Exploration of the Lungs by Means of Auscultation and Percussion," gave a unique illustration of the sounds obtained by percussion. He employed for the purpose a loaf of bread out of which he elicited the sounds peculiar to the various pathological changes occurring in the lung tissue. The normal vesicular resonance is reproduced in the tapping of a loaf of good bread. By soaking the loaf in water and thus filling up the interstices we simulate pulmonary œdema, and get the flat sound. If the absorption of water be not complete we get dulness proportioned to the degree of absorption. By substituting a solution of gelatine for water and allowing the bread to remain in it until the gelatine has congealed, we get a sound analogous to that occurring in the solidification of pneumonia. By introducing a solid substance into the loaf we may by percussion elicit the sounds brought out in deep solidification. Tympanitic resonance within a circumscribed space is illustrated by excavating a portion of the center of the loaf, leaving only the crust covering. By immersing the loaf for a few moments in water, the tympanitic resonance is brought into contrast with flatness in percussion. Circumscribed flatness may be shown by filling the space which gave the tympanitic resonance with some solid material, as dough containing no air, for instance.

To illustrate the vesiculo-tympanitic resonance remove from a loaf of bread by means of a hollow cylinder longitudinal sections in one half of the loaf. These spaces yield a tympanitic resonance and the portions of bread which remain give the vesicular sound. The tympanitic quality and pitch will correspond with the number of longitudinal sections removed.—*The Medical Age.*

---

Spray in Abdominal Surgery.—I have of late omitted the use of the spray in performing abdominal operations. For five years I have been an ardent, perhaps a bigoted, believer in the necessity for the use of the spray in operations involving the opening of the peritoneum; but having seen its disuse commenced by Keith, Tait, Bantock, and others, with equally good results as under its use, I have been unable to close my eyes to what has seemed to me to be the inexorable logic of facts. I would sum up the conclusions to which my observation has led me on this part of the subject by saying that increased operative experience and extreme cleanliness, in its fullest sense, are the two main factors which contribute to successful practice. This doctrine of cleanliness has doubtless been brought about very largely indeed, if not wholly, by the work which Lister has done; and I understand it to include many items, such as abundant use of water, most careful attention to sponging, arrest of hemorrhage, and drainage where necessary.

The points that have most strongly impressed me, and which I would impress also upon others, are that if there is the slightest doubt, before closing up the wound, as to the presence of fluid or the likelihood of much future oozing of bloody serum you must sponge very thoroughly indeed, and will probably require a drainage tube. Germs, even putrefactive germs, there probably may be often left behind, but I would leave behind as few as possible; and those that are left, I suppose, become innocuous, or are digested, or absorbed by the serous peritoneum without harm. provided that no fluid is allowed to be retained in the abdominal cavity, in which they would find a suitable soil for increase.—*Thomas Savage, M.D., in Birmingham Med. Review.*

---

Chloroform Water.—This is obtained by mixing three parts of water with one of chloroform, shaking the mixture well, and letting it stand for some time. The excess of chloroform is then removed by decantation, or is drawn off with a siphon.

The water thus obtained contains nine parts of chloroform to one hundred of water.

It has been ascertained that many salts, as chlorate of potash, bicarbonate of soda, salicylate of soda, etc., when dissolved in the water, do not prevent this solution of chloroform.

Lasegne and Regnault believe that the chloroform water thus obtained is the best preparation wherever the internal use of chloroform is required.

Chloroform water is very agreeable to taste, leaves in the mouth a sensation of coolness lasting for a few minutes after the water is swallowed. This water is well adapted as a vehicle for medicines having a disagreeable taste, its own peculiar taste generally acting as a complete disguise. For its local action chloroform water can be beneficially used in many affections of the mouth, gums, palate and pharynx.

The action of the water on the stomach differs according to the time taken.

Taken with wine it augments its stimulant properties. It is of efficacy in combatting digestive disturbances, acting as a local sedative as when in contact with the mucous surface of the mouth.

In conclusion, chloroform water is of benefit in dilatation of stomach and in cancer of stomach, quieting the pain arising from the digestive movements.—*Revista de Medicina.—Cincinnati Lancet and Clinic.*

---

MISTAKE BY A DRUGGIST—DAMAGES.—An action was brought against a druggist to recover $10,000 damages for causing the death of a pregnant wife and the child that was born. These deaths, it was alleged, were caused by a dose of sulphate of zinc, a deadly poison, given by the druggist in mistake for Epsom salts. A judgment was recovered for $1,000, and the defendant appealed the case —Walton *vs.* Booth—to the Supreme Court of Louisiana, which affirmed the judgment. The Chief Justice (Bermudez), in the opinion, said: "Though the death of the mother and child resulted from erysipelas, and not from the poison, from taking that the mother endured continued and great pain and suffering, for which she would have been entitled to recover. The defendant was greatly negligent. Druggists in the discharge of their functions should be required not only to be skillful, but also exceedingly cautious and prudent, in view of the terrific consequences which may attend, as they have not unfrequently in the past, the least inattention on their part. All persons who deal with deadly poisons are held to a strict responsibility for their use, and a druggist is undoubtedly held to a special degree of responsibility for the erroneous use of poisons, corresponding with his superior knowledge of their dispensation."—*Med. and Surg. Reporter.*

THE QUINTESSENCE OF ABSURDITY.—A novel dress has recently been exhibited at the rooms of the National Health Society, Berners street, London, says the *Medical Times and Gazette,* intended for the protection of sanitary visitors, nurses, and others who have to enter the apartments of persons suffering from infectious diseases. The garment is of mackintosh, glazed inside and out, and made to completely envelope the wearer, with a hood to cover the head. Only the hands and face remain exposed —a matter considered of little importance, as these can be easily washed with disinfectants. A not less important object proposed to be effected by the use of this dress is that by its removal when the wearer leaves the sick-room the clothes which have been protected need not be changed, and the danger of the disease being carried from house to house or communicated to susceptible people in public vehicles is obviated. A tight case for the fever-dress to be inclosed in is part of the invention. At the end of the day, or as often as may be convenient, the dress can be cleansed with disinfectants. Further protection is given by a simple form of respirator, which is made of two folds of washing-net, between which is placed a layer of medicated cotton-wool, through which the wearer can breathe, though no germs can pass. After use, the wool is burnt and the net washed.—*Louisville Med. News.*

---

DR. ROMANES tells a charming story of the father of the late Charles Darwin: "For the benefit of the district in which he lived, Dr. Darwin offered to dispense medicines gratis to any one who applied and was not able to pay. He was surprised to find that very few of the sick poor availed themselves of his offering, and, guessing that the reason must have been a dislike to become a recipient of charity, he devised a plan to neutralize this feeling. Whenever any poor persons applied for medical aid, he told them that he would supply the medicine, but that they must pay for the bottles. This little distinction made all the difference, and ever afterward the poor used to flock to the doctor's house for relief as a matter of right."—*Chicago Medical Review.*

